# Soft super-continuity and soft delta-closed graphs

**DOI:** 10.1371/journal.pone.0301705

**Published:** 2024-04-10

**Authors:** Dina Abuzaid, Samer Al Ghour, Monia Naghi

**Affiliations:** 1 Department of Mathematics, Faculty of Science, King Abdulaziz University, Jeddah, Saudi Arabia; 2 Department of Mathematics and Statistics, Jordan University of Science and Technology, Irbid, Jordan; TU Wien: Technische Universitat Wien, AUSTRIA

## Abstract

Introducing a strong form of soft continuity between soft topological spaces is significant because it can contribute to our growing understanding of soft topological spaces and their features, provide a basis for creating new mathematical tools and methods, and have significant applications in various fields. In this paper, we define soft super-continuity as a new form of soft mapping. We present various characterizations of this soft concept. Also, we show that soft super-continuity lies strictly between soft continuity and soft complete continuity and that soft super-continuity is a strong form of soft *δ*-continuity. In addition, we give some sufficient conditions for the equivalence between soft super-continuity and other related concepts. Moreover, we characterize soft semi-regularity in terms of super-continuity. Furthermore, we provide several results of soft composition, restrictions, preservation, and products by soft super-continuity. In addition to these, we study the relationship between soft super-continuity and soft *δ*-continuity with their analogous notions in general topology. Finally, we give several sufficient conditions on a soft mapping to have a soft *δ*-closed graph.

## 1 Introduction and preliminaries

Molodtsov [1] proposed soft sets as a beneficial alternative to existing mathematical methodologies for dealing with uncertainty. In contrast to probability theory, fuzzy set theory, and rough set theory, soft sets do not rely on precise values such as membership grade and probability. This is because, in most real cases, the genuine possibilities and membership grades are not well-known enough to assign precise values. This property of soft sets allows them to be used in various circumstances. Since its debut, the concept of soft sets has received significant attention and has been successfully used in various applications (for example, see [2–6]).

Several researchers have extended soft set theory to other mathematical structures, such as soft ideal theory [7], soft group theory [8], soft ring theory [9], soft *σ*-algebras [10, 11], and others.

Soft topology, which was presented in [12] as a merger of classical topology and soft set theory is one such structure. Noteworthy contributions to the development of soft topology in which authors modified and applied numerous traditional topological ideas to the setting of soft sets, for instance, soft compact spaces [13–15], soft metric [16], soft connected [17], soft separation axioms [18–22], and soft extremally disconnected spaces [23].

Both weak and strong kinds of soft open sets are essential in the study of soft topology because they provide a more general and flexible framework for investigating soft topological spaces and their features. They also provide a versatile framework for capturing uncertainty and vagueness in soft topology, allowing for variable levels of accuracy and ambiguity in diverse applications. Furthermore, they enable greater freedom in designing new classes of continuous functions, allowing us to construct functions that are better suited to specific applications and challenges. Therefore, this research area was and still is attractive to researchers. For instance, “soft semi-open” [24], “soft pre-open” [25], “soft *β*-open” [25], “soft *θ*-open” [26], “soft *α*-open” [27], “soft regular open” [28], “soft somewhere dense” [29], “soft *δ*-open” [30], “soft *ω*-open” [31], “weakly soft $\left(\tilde{\gamma },{\tilde{\gamma }}^{\prime }\right)$-open” [32], “soft *γ*-open” [33], “cluster soft sets” [34], “weakly soft *β*-open” [35], “weakly soft pre-open” [36], “soft parametric somewhat-open” [37], “weakly soft *α*-open” [38], and so on.

Soft continuity was defined as a main concept in the context of soft topological spaces [39]. Afterward, many forms of soft continuity appeared in the literature. For instance, “soft semi-continuous” [40], “soft pre-continuous” [27], “soft *β*-continuous” [41], “soft *θ*-continuous” [26], “soft *α*-continuous” [27], “soft *δ*-continuous” [30], “soft *ω*-continuous” [42], “soft *ω*_*s*_-continuous” [42], “weakly soft *β*-continuous” [35], “weakly soft pre-continuous” [36], “weakly soft *α*-continuous” [38], “soft complete continuous” [43], “soft strongly continuous” [43], “soft *C*-continuous” [44], “soft almost *C*-continuous” [44], “soft *ω*-*θ*-continuous” [45], “soft weakly *θ*_*ω*_-continuous” [45], and so on. The authors in [46] proved that soft continuity is useful in developing computational topological applications and digital images.

In this paper, we define soft super-continuity as a new form of soft mapping. We present various characterizations of this soft concept. Also, we show that soft super-continuity lies strictly between soft continuity and soft complete continuity and that soft super-continuity is a strong form of soft *δ*-continuity. In addition, we give some sufficient conditions for the equivalence between soft super-continuity and other related concepts. Moreover, we characterize soft semi-regularity in terms of super-continuity. Furthermore, we provide several results of soft composition, restrictions, preservation, and products by soft super-continuity. In addition, we study the relationship between each soft super-continuity soft *δ*-continuity and their analogous notions in general topology. Finally, we give several sufficient conditions on the soft mapping to have a soft *δ*-closed graph.

This article is organized as follows: In Section 2, we introduce the concept of “soft super-continuity,” a new type of soft mapping. We present several characterizations of them. Also, we study their relationships with some other soft continuity types. Moreover, we use them to characterize soft semi-regularity. Furthermore, we provide several results on soft composition, restrictions, preservation, and products related to soft super-continuity. In addition to these, we investigate the correspondence between soft super-continuity and soft *δ*-continuity and their analogous notions in general topology. In Section 3, we introduce several sufficient conditions for a soft mapping to have a soft *δ*-closed graph.

In the rest of this section, we introduce some basic definitions and terminology we will use in the sequel:

Let *E* be an initial universe and *Z* be a set of parameters. A soft set over *Z* relative to *E* is a function K:E→P(Z), where P(Z) is the power set of *Z*. The collection of soft sets over *E* relative to *Z* is denoted by *SS* (*Z*, *E*). Let *H* ∈ *SS* (*Z*, *E*). If *H* (*a*) = ∅ for each *a* ∈ *E*, then *H* is called the null soft set over *Z* relative to *E* and denoted by 0_*E*_. If *H* (*a*) = *Z* for all *a* ∈ *E*, then *H* is called the absolute soft set over *Z* relative to *E* and denoted by 1_*E*_. If there exist *b* ∈ *E* and *y* ∈ *Z* such that *H* (*b*) = {*y*} and *H* (*a*) = ∅ for all *a* ∈ *E* − {*b*}, then *H* is called a soft point over *E* relative to *Z* and denoted by *b*_*y*_. The collection of all soft points over *E* relative to *Z* is denoted by *SP* (*Z*, *E*). If for some *b* ∈ *E* and *X* ⊆ *Z*, *H* (*b*) = *X* and *H* (*a*) = ∅ for all *a* ∈ *L* − {*b*}, then *K* will be denoted by *a*_*X*_. If for some *X* ⊆ *Z*, *H* (*a*) = *X* for all *a* ∈ *E*, then *H* will be denoted by *C*_*X*_. If *H* ∈ *SS* (*Z*, *E*) and *a*_*x*_ ∈ *SP* (*Z*, *E*), then *a*_*x*_ is said to belong to *H* (notation: $a_{x}\tilde{\in }H$) if *x* ∈ *H* (*a*). Soft topological spaces were defined in [12] as follows: A triplet (Z,ℜ,E), where ℜ⊆SS(Z,E), is called a soft topological space if 0_*E*_, 1_*A*_ ∈ ℜ, and ℜ is closed under finite soft intersections and arbitrary soft unions.

Throughout this paper, we will use concepts and phrases as they appear in [31, 47].

Let (Z,ℜ,E) be a soft topological space, (*Z*, *ξ*) be a topological space, *M* ∈ *SS* (*Z*, *E*), and *A* ⊆ *Z*. Then $Int_{ℜ}\left(M\right)$, $Cl_{ℜ}\left(M\right)$, *Int*_*ξ*_(*A*), and *Cl*_*ξ*_(*A*), ${ℜ}^{c}$, and *ξ*^*c*^ will denote the soft interior of *M* in (Z,ℜ,E), the soft closure of *M* in (Z,ℜ,E), the interior of *A* in (*Z*, *ξ*), and the closure of *A* in (*Z*, *ξ*), the collection of all soft closed sets in (Z,ℜ,E), and the family of all closed sets in (*Z*, *ξ*).

**Definition 1.1**. *Let* (*Z*, *ξ*) *be a topological space, and let* *A* ⊆ *Z*. *Then* *A* *is called a*

*(a)* [48] “*regular open set in* (*Z*, *ξ*)” *if A* = *Int*_*ξ*_(*Cl*_*ξ*_(*A*)). *RO* (*ξ*) *will denote the collection of all regular open sets in* (*Z*, *ξ*).
*(b)* [49] “*δ*-*open set in* (*Z*, *ξ*)” *if for each z* ∈ *A*, *we find U* ∈ λ *such that z* ∈ *U* ⊆ *Int*_*ξ*_(*Cl*_*ξ*_(*U*)) ⊆ *A*. *ξ*_*δ*_ *will denote the collection of all δ-open sets in* (*Z*, *ξ*).

It is well known that *ξ*_*δ*_ is a topology having *RO* (*ξ*) as a base.

**Definition 1.2**. *A mapping g* : (*Z*, *ξ*) → (*Z*, *ϕ*) *is called*

*(a)* [50] “*δ-continuous” if for every z* ∈ *Z and V* ∈ *RO* (*ϕ*) *such that g*(*z*) ∈ *V*, *we find W* ∈ *RO* (*ξ*) *such that* *z* ∈ *W and g*(*W*) ⊆ *V*.
*(b)* [51] “*super-continuous (Notation: SC)” if g*^−1^(*V*) ∈ *ξ*_*δ*_ *for every V* ∈ *ϕ*.

**Definition 1.3**. *Let* (Z,ℜ,E) *be a soft topological space, and let K* ∈ *SS* (*Z*, *E*). *Then K is called a*

*(a)* [28] “*soft regular open set in* (Z,ℜ,E)” *if* $K=Int_{ℜ}\left(Cl_{ℜ}\left(K\right)\right)$. *The soft complement of a soft δ-open set in* (Z,ℜ,E) *is called a “soft* *δ*-*closed set in* (Z,ℜ,E)”. RO(ℜ) (*resp*. RC(ℜ)) *will denote the collection of all soft regular open (resp. soft regular open) sets in* (Z,ℜ,E).
*(b)* [30] “*soft δ-open set in* (Z,ℜ,E)” *if for each* $e_{z}\tilde{\in }K$, *we find H* ∈ ℜ *such that* $e_{z}\tilde{\in }H\tilde{\subseteq }Int_{ℜ}\left(Cl_{ℜ}\left(H\right)\right)\tilde{\subseteq }K$. ℜ_*δ*_ *will denote the collection of all δ*-*open sets in* (Z,ℜ,E).

It is well known that ℜ_*δ*_ is a soft topology having RO(ℜ) as a soft base.

**Definition 1.4**. *A soft mapping* $f_{qv}:(Z,ℜ,E)\to (Y,P,F)$ *is called*

*(a)* [26] “*soft θ-continuous” if for each* $e_{z}\tilde{\in }SS\left(Z,E\right)$ *and each G* ∈ ℘ *such that* $f_{qv}\left(e_{z}\right)\tilde{\in }G$, *there exists* *K* ∈ ℜ *such that* $e_{z}\tilde{\in }K$ *and* $f_{qv}\left(Cl_{ℜ}\left(K\right)\right)\tilde{\subseteq }Cl_{P}\left(G\right)$.
*(b)* [30] “*soft δ-continuous” if for each* $e_{z}\tilde{\in }SS\left(Z,E\right)$ *and each G* ∈ *RO* (℘) *such that* $f_{qv}\left(e_{z}\right)\tilde{\in }G$, *there exists* K∈RO(ℜ) *such that* $e_{z}\tilde{\in }K$ *and* $f_{qv}\left(Cl_{ℜ}\left(K\right)\right)\tilde{\subseteq }Cl_{P}\left(G\right)$.
*(c)* [52] “*soft almost continuous*” *if for each* $e_{z}\tilde{\in }SS\left(Z,E\right)$ *and each G* ∈ ℘ *such that* $f_{qv}\left(e_{z}\right)\tilde{\in }G$, *there exists K* ∈ ℜ *such that* $e_{z}\tilde{\in }K$ *and* $f_{qv}\left(K\right)\tilde{\subseteq }Int_{P}(Cl_{P}\left(G\right))$.
*(d)* [53] “*soft almost open” if f*_*qv*_(*K*) ∈ ℘ *for every* K∈RO(ℜ).
*(e)* [43] “*soft complete continuous” if* $f_{qv}^{-1}\left(G\right)\in RO(ℜ)$ *for every G* ∈ ℘.

**Definition 1.5**. *A soft topological space* (Z,ℜ,E) *is said to be*

*(a)* [53] “*soft Hausdorff” if for every two soft points* *e*_*x*_, *s*_*y*_ ∈ *SP* (*Z*, *E*) *with* *e*_*x*_ ≠ *s*_*y*_, *there exist* *M*, *N* ∈ ℜ *such that* $e_{x}\tilde{\in }M$, $s_{y}\tilde{\in }N$, *and* $M\tilde{\cap }N=0_{E}$.
*(b)* [53] “*soft regular*” *if for every e*_*z*_ ∈ *SP* (*Z*, *E*) *and every T* ∈ ℜ *such that* $e_{z}\tilde{\in }ℜ$, *there exists K* ∈ ℜ *such that* $a_{z}\tilde{\in }K\tilde{\subseteq }Cl_{ℜ}(K)\tilde{\subseteq }T$.
*(c)* [54] “*soft semi-regular*” *if for each* $e_{z}\tilde{\in }SS\left(Z,E\right)$ *and each* *K* ∈ ℜ, *there exists* G∈RO(ℜ) *such that* $e_{z}\tilde{\in }G\tilde{\subseteq }K$.
*(d)* [55] “*soft almost regular*” *if for each* K∈RC(ℜ) *and each* $e_{z}\tilde{\in }1_{E}-K$, *there are* *M*, *N* ∈ ℜ *such that* $e_{z}\tilde{\in }M$, $K\tilde{\subseteq }N$, *and* $M\tilde{\cap }N=0_{E}$.
*(e)* [56] “*soft nearly compact*” (*resp. “soft nearly Lindelof”*) *if for each* H⊆RO(ℜ) *such that* ${\tilde{\cup }}_{H\in H}H=1_{E}$, *there exists a finite (resp. countable) subcollection* $H_{1}\subseteq H$ such ${\tilde{\cup }}_{H\in H_{1}}H=1_{E}$.

**Definition 1.6**. [56] *Let* (Z,ℜ,E) *be a soft topological space, and let* *K* ∈ *SS* (*Z*, *E*). *Then K is called a “soft nearly compact set relative to* (Z,ℜ,E)” (*resp. “soft nearly Lindelof set relative to* (Z,ℜ,E)”) *if for each* H⊆RO(ℜ) *such that* $K\tilde{\subseteq }{\tilde{\cup }}_{H\in H}H$, *there exists a finite (resp. countable) subcollection* $H_{1}\subseteq H$ *such* $K\tilde{\subseteq }{\tilde{\cup }}_{H\in H_{1}}H$.

**Definition 1.7**. [13] *Let* (Z,ℜ,E) *and* (*Y*, ℘, *F*) *be two soft topological spaces, and let* B={M×N:M∈ℜandN∈℘}. *Then the soft topology over* *Z* × *Y* *relative to* *E* × *F* *having* B *as a soft base is called the product soft topology and is denoted by* pr(ℜ×P).

## 2 Soft super-continuity

In this section, we introduce the concept of “soft super-continuity,” a new type of soft mapping. We present several characterizations of them. Also, we study their relationships with some other soft continuity types. Moreover, we use them to characterize soft semi-regularity. Furthermore, we provide several results on soft composition, restrictions, preservation, and products related to soft super-continuity. In addition to these, we investigate the correspondence between soft super-continuity and soft *δ*-continuity and their analogous notions in general topology.

**Definition 2.1**. *A soft mapping* $f_{qv}:(Z,ℜ,E)\to (Y,P,F)$ *is called soft super-continuous (notation: SC) if* $f_{qv}^{-1}\left(K\right)\in$ ℜ_*δ*_ *for every K* ∈ ℘.

The following result gives several characterizations of soft super-continuous mappings:

**Theorem 2.2**. *For a soft mapping* $f_{qv}:(Z,ℜ,E)\to (Y,P,F)$, *the following are equivalent*:

(1)
$f_{qv}:(Z,ℜ,E)\to (Y,P,F)$ *is soft SC*.
(2)
$f_{qv}^{-1}(T)\in {\left({ℜ}_{\delta }\right)}^{c}$ *for every* *T* ∈ ℘^*c*^.
(3)
$Cl_{{ℜ}_{\delta }}(f_{qv}^{-1}(A))\tilde{\subseteq }f_{qv}^{-1}(Cl_{P}(A))$ *for each* A∈SS(Y,F) .
(4)
$f_{qv}^{-1}(Int_{P}(A))\tilde{\subseteq }Int_{{ℜ}_{\delta }}(f_{qv}^{-1}(A))$ *for each* A∈SS(Y,F) .
(5) *f*_*qv*_ : (*Z*, ℜ_*δ*_, *E*) → (*Y*, ℘, *F*) *is soft continuous*.
(6) *For a soft base* B *for* (*Y*, ℘, *F*), $f_{qv}^{-1}(B)\in {ℜ}_{\delta }$ *for every* B∈B.
(7) *For a soft subbase* S *for* (*Y*, ℘, *F*), $f_{qv}^{-1}(S)\in {ℜ}_{\delta }$ *for every* S∈S.
(8) *For each* *e*_*z*_ ∈ *SP* (*Z*, *E*) *and each* *G* ∈ ℘ *such that* $f_{qv}(e_{z})\tilde{\in }G$, *we find H* ∈ ℜ_*δ*_ *such that* $e_{z}\tilde{\in }H$ *and* $f_{qv}(H)\tilde{\subseteq }G$.
(9) *For each* *e*_*z*_ ∈ *SP* (*Z*, *E*) *and each* *G* ∈ ℘ *such that* $f_{qv}(e_{z})\tilde{\in }G$, *we find* *K* ∈ ℜ *such that* $e_{z}\tilde{\in }K$ *and* $f_{qv}(Int_{ℜ}(Cl_{ℜ}(K)))\tilde{\subseteq }G$.

*Proof*. (1) → (2): Let *T* ∈ ℘^*c*^. Then 1_*F*_ − *T* ∈ ℘. So, by (1), $f_{qv}^{-1}(1_{F}-T)=1_{E}-f_{qv}^{-1}(T)\in {ℜ}_{\delta }$. Hence, $f_{qv}^{-1}(T)\in {\left({ℜ}_{\delta }\right)}^{c}$.

(2) → (3): Let A∈SS(Y,F). Then *Cl*_℘_(*A*) ∈ ℘^*c*^. So, by (2), $f_{qv}^{-1}(Cl_{P}(A))\in {\left({ℜ}_{\delta }\right)}^{c}$. Since $f_{qv}^{-1}(A)\tilde{\subseteq }f_{qv}^{-1}(Cl_{P}(A))\in {\left({ℜ}_{\delta }\right)}^{c}$, then $Cl_{{ℜ}_{\delta }}(f_{qv}^{-1}(A))\tilde{\subseteq }f_{qv}^{-1}(Cl_{P}(A))$.

(3) → (4): Let A∈SS(Y,F). Then, by (3),

$$\begin{array}{ccc} 1_{E}-Int_{{ℜ}_{\delta }}(f_{qv}^{-1}(A)) & = & Cl_{{ℜ}_{\delta }}(1_{E}-f_{qv}^{-1}(A))\\ & = & Cl_{{ℜ}_{\delta }}(f_{qv}^{-1}(1_{F}-A))\\ & \tilde{\subseteq } & f_{qv}^{-1}(Cl_{P}(1_{F}-A))\\ & = & f_{qv}^{-1}(1_{F}-Int_{P}(A))\\ & = & 1_{E}-f_{qv}^{-1}(Int_{P}(A)) \end{array}$$

and so $f_{qv}^{-1}(Int_{P}(A))\tilde{\subseteq }Int_{{ℜ}_{\delta }}(f_{qv}^{-1}(A))$.

(4) → (5): Let *K* ∈ ℘. Then *Int*_℘_(*K*) = *K*, and by (4), $f_{qv}^{-1}(K)\tilde{\subseteq }Int_{{ℜ}_{\delta }}(f_{qv}^{-1}(K))$. Thus, $f_{qv}^{-1}(K)=Int_{{ℜ}_{\delta }}(f_{qv}^{-1}(K))$. Hence, $f_{qv}^{-1}(K)\in {ℜ}_{\delta }$. This shows that *f*_*qv*_ : (*Z*, ℜ_*δ*_, *E*) → (*Y*, ℘, *F*) is soft continuous.

(5) → (6) and (6) → (7) are obvious.

(7) → (8): Let *K* ∈ ℘. To show that $f_{qv}^{-1}\left(K\right)\in {ℜ}_{\delta }$, let $e_{z}\tilde{\in }f_{qv}^{-1}\left(K\right)$. Then we find $S_{1},S_{2},...,S_{n}\in S$ such that $f_{qv}(e_{z})\tilde{\in }S_{1}\tilde{\cap }S_{2}\tilde{\cap }...\tilde{\cap }S_{n}\tilde{\subseteq }K$ and so $e_{z}\tilde{\in }f_{qv}^{-1}(S_{1})\tilde{\cap }f_{qv}^{-1}(S_{2})\tilde{\cap }...\tilde{\cap }f_{qv}^{-1}(S_{n})\tilde{\subseteq }f_{qv}^{-1}\left(K\right)$. Let $G=f_{qv}^{-1}(S_{1})\tilde{\cap }f_{qv}^{-1}(S_{2})\tilde{\cap }...\tilde{\cap }f_{qv}^{-1}(S_{n})$. Then $e_{z}\tilde{\in }G$, $f_{qv}(G)\tilde{\subseteq }K$, and by (7), *G* ∈ ℜ_*δ*_. This ends the proof.

(8) → (9): Let *e*_*z*_ ∈ *SP* (*M*, *Z*) and *G* ∈ ℘. Then, by (8), *H* ∈ ℜ_*δ*_ such that $e_{z}\tilde{\in }H$ and $f_{qv}(H)\tilde{\subseteq }G$. Choose K∈RO(ℜ) such that $e_{z}\tilde{\in }K=Int_{ℜ}(Cl_{ℜ}(K))\tilde{\subseteq }H$. Therefore, we have $e_{z}\tilde{\in }K\in ℜ$ and $f_{qv}(Int_{ℜ}(Cl_{ℜ}(K)))\tilde{\subseteq }f_{qv}(H)\tilde{\subseteq }G$.

(9) → (1): Let *G* ∈ ℘ and let $e_{z}\tilde{\in }f_{qv}^{-1}(G)$. Then *f*_*qv*_ (*e*_*z*_) $\tilde{\in }G$, and by (9), there exists *K* ∈ ℜ such that $e_{z}\tilde{\in }K$ and $f_{qv}(Int_{ℜ}(Cl_{ℜ}(K)))\tilde{\subseteq }G$. Then we have $Int_{ℜ}(Cl_{ℜ}(K))\in {ℜ}_{\delta }$ and $e_{z}\tilde{\in }K\tilde{\subseteq }Int_{ℜ}(Cl_{ℜ}(K))\tilde{\subseteq }f_{qv}^{-1}(f_{qv}(Int_{ℜ}(Cl_{ℜ}(K))))\tilde{\subseteq }f_{qv}^{-1}(G)$. This shows that $f_{qv}^{-1}(G)\in {ℜ}_{\delta }$.

**Theorem 2.3**. *If* $f_{qv}:(Z,ℜ,E)\to (Y,P,F)$ *is soft SC, then* $q:(Z,{ℜ}_{a})\to (Y,P_{v(a)})$ *is SC for every a* ∈ *E*.

*Proof*. Suppose that $f_{qv}:(Z,ℜ,E)\to (Y,P,F)$ is soft SC, and let *a* ∈ *E*. By Theorem 4.9(5), *f*_*qv*_ : (*Z*, ℜ_*δ*_, *E*) → (*Y*, ℘, *F*) is soft continuous. So, by Proposition 3.8 of [58], *q* : (*Z*, (ℜ_*δ*_)_*a*_) → (*Y*, ℘_*v*(*a*)_) is continuous. Since by Theorem 30 of [57], ${\left({ℜ}_{\delta }\right)}_{a}={\left({ℜ}_{a}\right)}_{\delta }$, then $q:(Z,{\left({ℜ}_{a}\right)}_{\delta })\to (Y,P_{v(a)})$ is continuous. Hence, $q:(Z,{ℜ}_{a})\to (Y,P_{v(a)})$ is SC.

The following two results discuss the relationships between soft super-continuity and its analogous concept in general topology:

**Theorem 2.4**. *Let* {(*Z*, *β*_*e*_) : *e* ∈ *E*} *and* {(*Y*, *α*_*f*_) : *f* ∈ *F*} *be two collections of TSs. Let q* : *Z* → *Y* *and v* : *E* → *F* *be mappings where v is bijective. Then f*_*qv*_ : (*Z*, ⊕_*e*∈*E*_*β*_*e*_, *E*) → (*Y*, ⊕_*f*∈*F*_*α*_*f*_, *F*) *is soft SC if and only if* *q* : (*Z*, *β*_*e*_) → (*Y*, *α*_*v*(*e*)_) *is SC for all e* ∈ *E*.

*Proof. Necessity*. Let *f*_*qv*_ : (*Z*, ⊕_*e* ∈ *E*_*β*_*e*_, *E*) → (*Y*, ⊕_*f* ∈ *F*_*α*_*f*_, *F*) be soft SC. Let *e* ∈ *E*. Then, by Theorem 2.3, *q* : (*Z*, (⊕_*e*∈*E*_*β*_*z*_)_*e*_) → (*Y*, (⊕_*f*∈*F*_*α*_*f*_)_*v*(*e*)_) is SC. But by Theorem 3.11 of [47], (⊕_*e* ∈ *E*_*β*_*e*_)_*e*_ = *β*_*e*_ and (⊕_*f*∈*F*_*α*_*f*_)_*v*(*e*)_ = *α*_*v*(*e*)_. Hence, *q* : (*Z*, *β*_*e*_) → (*Y*, *α*_*v*(*e*)_) is SC.

*Sufficiency.* Let *q* : (*Z*, *β*_*e*_) → (*Y*, *α*_*v*(*e*)_) be SC for all *e* ∈ *E*. Let *K* ∈ ⊕_*f* ∈ *F*_*α*_*f*_. By Theorem 31 of [57], it is sufficient to show that $(f_{qv}^{-1}\left(K\right))(e)\in {\left(\beta_{e}\right)}_{\delta }$ for all *e* ∈ *E*. Let *e* ∈ *E*. Since *q* : (*Z*, *β*_*e*_) → (*Y*, *α*_*v*(*e*)_) is SC and *K* (*v*(*e*)) ∈ *α*_*v*(*e*)_, then $(f_{qv}^{-1}\left(K\right))(e)=q^{-1}(K(v(e)))\in {\left(\beta_{e}\right)}_{\delta }$.

**Corollary 2.5**. *Let q* : (*Z*, *ξ*) → (*Y*, *ϕ*) *and v* : *E* → *F* *be two mappings where v is a bijection. Then q* : (*Z*, *ξ*) → (*Y*, *ϕ*) *is SC if and only if f*_*qv*_ : (*Z*, *τ*(*ξ*), *E*) → (*Y*, *τ*(*ϕ*), *F*) *is soft SC*.

*Proof*. For each *e* ∈ *E* and *f* ∈ *F*, put *β*_*e*_ = *ξ* and *α*_*f*_ = *ϕ*. Then *τ*(*α*) = ⊕_*e*∈*E*_*β*_*e*_ and *τ*(*ϕ*) = ⊕_*f*∈*F*_*α*_*f*_. By using Theorem 2.4, we get the result.

In Theorem 2.6 and Example 2.7, we discuss the relationships between the classes of soft SC mappings and soft continuous mappings:

**Theorem 2.6**. *Every soft SC mapping is soft continuous*.

*Proof*. Let $f_{qv}:(Z,ℜ,E)\to (Y,P,F)$ be soft SC. Let *K* ∈ ℘. Then, by Theorem 2.2(5), $f_{qv}^{-1}\left(K\right)\in {ℜ}_{\delta }\subseteq ℜ$. Hence, *f*_*qv*_ is soft continuous.

Theorem 2.6 is not reversible.

**Example 2.7**. *Let* Z=R, *E* = {*a*, *b*, *d*}, *and* $ℜ=\{0_{E},1_{E},a_{\left(0,1\right)}\}$. *Suppose that* $Int_{{ℜ}_{\delta }}(a_{\left(0,1\right)})\neq 0_{E}$. *Then we find x* ∈ (0, 1) *such that* $a_{x}\tilde{\in }Int_{{ℜ}_{\delta }}(a_{\left(0,1\right)})$. *So, we find K* ∈ ℜ *such that* $a_{x}\tilde{\in }K\tilde{\subseteq }Int_{ℜ}(Cl_{ℜ}(K))\tilde{\subseteq }a_{\left(0,1\right)}$. *Thus, K* = *a*_(0,1)_, *and so* $Int_{ℜ}(Cl_{ℜ}(K))=Int_{ℜ}(1_{E})=1_{E}\tilde{\subseteq }a_{\left(0,1\right)}$. *Hence*, $Int_{{ℜ}_{\delta }}(a_{\left(0,1\right)})=0_{E}$. *Let q* : *Z* → *Z* and *v* : *E* → *E be the identity mappings. Since* $f_{qv}^{-1}(a_{\left(0,1\right)})=a_{\left(0,1\right)}\in ℜ-{ℜ}_{\delta }$, *then* $f_{qv}:(Z,ℜ,E)\to (Z,ℜ,E)$ *is soft continuous but not soft SC*.

In Theorem 2.8 and Example 2.9, we discuss the relationships between the classes of soft complete continuous mappings and soft SC mappings:

**Theorem 2.8**. *Every soft complete continuous mapping is soft SC*.

*Proof*. Let $f_{qv}:(Z,ℜ,E)\to (Y,P,F)$ be soft complete continuous. Let *K* ∈ ℘. Then $f_{qv}^{-1}\left(K\right)\in RO(ℜ)\subseteq {ℜ}_{\delta }$. Hence, *f*_*qv*_ is soft SC.

Theorem 2.8 is not reversible.

**Example 2.9**. *Let Z* = {1, 2, 3}, *ξ* = {∅, *Z*, {1}, {3}, {1, 3}}, *and E* = {*a*, *b*}. *Consider the identity mappings q* : (*Z*, *ξ*) → (*Z*, *ξ*) *and v* : *E* → *E*. *It is not difficult to see that RO* (*ξ*) = {∅, *Z*, {1}, {3}}. *Then* *q* *is SC. On the other hand, since* {1, 3} ∈ *ξ* *while q*^−1^({1, 3}) = {1, 3} ∉ *RO* (*ξ*), *then q is not complete continuous. Therefore, by Corollary 2.5 and Corollary 1 of* [43], *f*_*qv*_ : (*Z*, *τ*(*ξ*), *E*) → (*Z*, *τ*(*ξ*), *E*) *is soft SC but not soft complete continuous*.

**Theorem 2.10**. *If* $f_{qv}:(Z,ℜ,E)\to (Y,P,F)$ *is soft δ-continuous, then* $q:(Z,{ℜ}_{a})\to (Y,P_{v(a)})$ *is δ-continuous for every a* ∈ *E*.

*Proof*. Suppose that $f_{qv}:(Z,ℜ,E)\to (Y,P,F)$ is soft *δ*-continuous. Then, by Theorem 6.2(6) of [30], *f*_*qv*_ : (*Z*, ℜ_*δ*_, *E*) → (*Y*, ℘_*δ*_, *F*) is soft continuous. So, by Proposition 3.8 of [58], *f*_*qv*_ : (*Z*, (ℜ_*δ*_)_*a*_) → (*Y*, (℘_*δ*_)_*v*(*a*)_) is soft continuous. Since by Theorem 30 of [57], ${\left({ℜ}_{\delta }\right)}_{a}={\left({ℜ}_{a}\right)}_{\delta }$ and (℘_*δ*_)_*v*(*a*)_ = (℘_*v*(*a*)_)_*δ*_, then $q:(Z,{\left({ℜ}_{a}\right)}_{\delta })\to (Y,{\left(P_{v(a)}\right)}_{\delta })$ is continuous. Hence, by Theorem 2.2(7) of [50], $q:(Z,{ℜ}_{a})\to (Y,P_{v(a)})$ is *δ*-continuous.

The following two results discuss the relationships between soft *δ*-continuity and its analogous concept in general topology:

**Theorem 2.11**. *Let* {(*Z*, *β*_*e*_) : *e* ∈ *E*} *and* {(*Y*, *α*_*f*_) : *f* ∈ *F*} *be two collections of TSs. Let q* : *Z* → *Y* *and v* : *E* → *F* *be mappings where v is bijective. Then f*_*qv*_ : (*Z*, ⊕_*e* ∈ *E*_*β*_*e*_, *E*) → (*Y*, ⊕_*f* ∈ *F*_*α*_*f*_, *F*) *is soft* *δ-continuous if and only if q* : (*Z*, *β*_*e*_) → (*Y*, *α*_*v*(*e*)_) *is* *δ-continuous for all e* ∈ *E*.

*Proof. Necessity*. Let *f*_*qv*_ : (*Z*, ⊕_*e* ∈ *E*_*β*_*e*_, *E*)→ (*Y*, ⊕_*f* ∈ *F*_*α*_*f*_, *F*) be soft *δ*-continuous. Let *e* ∈ *E*. Then by Theorem 2.10, *q* : (*Z*, (⊕_*e* ∈ *E*_*β*_*z*_)_*e*_)→ (*Y*, (⊕_*f* ∈ *F*_*α*_*f*_)_*v*(*e*)_) is *δ*-continuous. But by Theorem 3.11 of [47], (⊕_*e* ∈ *E*_*β*_*e*_)_*e*_ = *β*_*e*_ and (⊕_*f* ∈ *F*_*α*_*f*_)_*v*(*e*)_ = *α*_*v*(*e*)_. Hence, *q* : (*Z*, *β*_*e*_) → (*Y*, *α*_*v*(*e*)_) is *δ*-continuous.

*Sufficiency.* Let *q* : (*Z*, *β*_*e*_) → (*Y*, *α*_*v*(*e*)_) be *δ*-continuous for all *e* ∈ *E*. Let *K* ∈ (⊕_*f* ∈ *F*_*α*_*f*_)_*δ*_. Then by Theorem 31 of [57], *K* (*f*) ∈ (*α*_*f*_)_*δ*_ for all *f* ∈ *F*. By Theorem 31 of [57] and Theorem 6.2(7) of [30], it is sufficient to show that $(f_{qv}^{-1}\left(K\right))(e)\in {\left(\beta_{e}\right)}_{\delta }$ for all *e* ∈ *E*. Let *e* ∈ *E*. Since *q* : (*Z*, *β*_*e*_) → (*Y*, *α*_*v*(*e*)_) is *δ*-continuous and *K* (*v*(*e*)) ∈ (*α*_*v*(*e*)_)_*δ*_, then $(f_{qv}^{-1}\left(K\right))(e)=q^{-1}(K(v(e)))\in {\left(\beta_{e}\right)}_{\delta }$.

**Corollary 2.12**. *Let q* : (*Z*, *ξ*) → (*Y*, *ϕ*) *and v* : *E* → *F* *be two mappings where v is a bijection. Then q* : (*Z*, *ξ*) → (*Y*, *ϕ*) *is* *δ-continuous if and only if* *f*_*qv*_ : (*Z*, *τ*(*ξ*), *E*) → (*Y*, *τ*(*ϕ*), *F*) *is soft δ-continuous*.

*Proof*. For each *e* ∈ *E* and *f* ∈ *F*, put *β*_*e*_ = *ξ* and *α*_*f*_ = *ϕ*. Then *τ*(*α*) = ⊕_*e* ∈ *E*_*β*_*e*_ and *τ*(*ϕ*) = ⊕_*f*∈*F*_*α*_*f*_. By using Theorem 2.11, we get the result.

In Theorem 2.13 and Example 2.14, we discuss the relationships between the classes of soft SC mappings and soft *δ*-continuous mappings:

**Theorem 2.13**. *Every soft SC mapping is soft* *δ-continuous*.

*Proof*. Let $f_{qv}:(Z,ℜ,E)\to (Y,P,F)$ be soft SC. Let *G* ∈ *RO* (℘) ⊆ ℘. Then $f_{qv}^{-1}(G)\in {ℜ}_{\delta }$. Thus, by Theorem 6.2(7) of [30], *f*_*qv*_ soft *δ*-continuous.

Theorem 2.13 is not reversible.

**Example 2.14**. *Let* Z=R, *ξ* *the usual topology on Z*, *ϕ* *the co-countable topology on Z, and E* = {*a*, *b*}. *Consider the identity mappings q* : (*Z*, *ξ*) → (*Z*, *ϕ*) *and v* : *E* → *E*. *Since RO* (*ϕ*) = {∅, *Z*}, *then q is δ-continuous. On the other hand, since* R-Q∈ϕ *while* $q^{-1}(R-Q)=R-Q\notin \xi_{\delta }$, *then q is not SC. Therefore, by Corollaries 2.5 and 2.12, f*_*qv*_ : (*Z*, *τ*(*ξ*), *E*) → (*Z*, *τ*(*ϕ*), *F*) *is soft δ-continuous but not soft SC*.

The following result provides sufficient conditions for the equivalence between soft SC mappings and soft *δ*-continuous mappings:

**Theorem 2.15**. *If* $f_{qv}:(Z,ℜ,E)\to (Y,P,F)$ *is a soft δ-continuous mapping such that* (*Y*, ℘, *F*) *is soft semi-regular, then f*_*qv*_ *is soft SC*.

*Proof*. Let *f*_*qv*_ be soft *δ*-continuous such that (*Y*, ℘, *F*) is soft semi-regular. Let *e*_*z*_ ∈ *SS* (*Z*, *E*) and let *G* ∈ ℘ such that $f_{qv}(e_{z})\tilde{\in }G$. Since (*Y*, ℘, *F*) is soft semi-regular, then there exists *K* ∈ ℘ such that $f_{qv}(e_{z})\tilde{\in }K\tilde{\subseteq }Int_{P}\left(Cl_{P}(K)\right)\tilde{\subseteq }G$. Since *f*_*qv*_ is a soft *δ*-continuous, then there exists *H* ∈ ℜ such that $e_{z}\tilde{\in }H$ and $f_{qv}(Int_{ℜ}\left(Cl_{ℜ}(H)\right))\tilde{\subseteq }Int_{P}\left(Cl_{P}(K)\right)\tilde{\subseteq }G$. This shows that *f*_*qv*_ is soft SC.

Soft semi-regularity condition cannot be removed from Theorem 2.15, as it can be deduced from Example 2.14.

**Corollary 2.16**. *If* $f_{qv}:(Z,ℜ,E)\to (Y,P,F)$ *is a soft δ-continuous mapping such that* (*Y*, ℘, *F*) *is soft regular, then f*_*qv*_ *is soft SC*.

The following result introduces a new characterization of soft semi-regularity:

**Theorem 2.17**. *The following are equivalent for any STS* (Z,ℜ,E).

*(a)* (Z,ℜ,E) *is soft semi-regular*.
*(b) Every soft continuous mapping* $f_{qv}:(Z,ℜ,E)\to (Y,P,F)$ *is also soft SC*.

*Proof*. (a) ⇒ (b): Let $f_{qv}:(Z,ℜ,E)\to (Y,P,F)$ be soft continuous. By (a), ${ℜ}_{\delta }=ℜ$. Thus, *f*_*qv*_ : (*Z*, ℜ_*δ*_, *E*) → (*Y*, ℘, *F*) is soft continuous. Hence, by Theorem 2.2(5), $f_{qv}:(Z,ℜ,E)\to (Y,P,F)$ is also soft SC.

(b) ⇒ (a): We will show that $ℜ\subseteq {ℜ}_{\delta }$. Let *G* ∈ ℜ. Take (Y,P,F)=(Z,ℜ,E) and *f*_*qv*_ the identity soft mapping (Z,ℜ,E). Since $f_{qv}:(Z,ℜ,E)\to (Z,ℜ,E)$ is soft continuous, then by (b), $f_{qv}:(Z,ℜ,E)\to (Z,ℜ,E)$ is soft SC. Thus, by Theorem 2.2(5), $f_{qv}^{-1}\left(G\right)=G\in {ℜ}_{\delta }$. This shows that $ℜ\subseteq {ℜ}_{\delta }$.

**Corollary 2.18**. *If* $f_{qv}:(Z,ℜ,E)\to (Y,P,F)$ *is a soft δ-continuous mapping such that* (Z,ℜ,E) *and* (*Y*, ℘, *F*) *are both soft semi-regular, then the following are equivalent*:

*(a) f*_*qv*_ *is soft SC*.
*(b) f*_*qv*_ *is soft δ-continuous*.
*(c) f*_*qv*_ *is soft continuous*.

*Proof*. Follows from Theorems 2.15 and 2.17.

**Corollary 2.19**. *If* $f_{qv}:(Z,ℜ,E)\to (Y,P,F)$ *is a soft continuous mapping such that* (Z,ℜ,E) *is soft regular, then f*_*qv*_ *is soft SC*.

*Proof*. The proof follows from Theorem 2.17.

In Theorems 2.20, 2.21, and 2.22, we discuss the behavior of soft SC mappings under soft composition:

**Theorem 2.20**. *If* $f_{q_{1}v_{1}}:(Z,ℜ,E)\to (Y,P,F)$ *is soft SC and* $f_{q_{2}v_{2}}:(Y,P,F)\to (W,ℜ,L)$ *is soft continuous, then* $f_{\left(q_{2}\circ q_{1})(v_{2}\circ v_{1}\right)}$ *is soft SC*.

*Proof*. Let $f_{q_{1}v_{1}}$ be soft SC and $f_{q_{2}v_{2}}$ be soft continuous. Let *K* ∈ ℜ. Since $f_{q_{2}v_{2}}$ is soft continuous, $f_{q_{2}v_{2}}^{-1}(K)\in P$. Since $f_{q_{1}v_{1}}$ is soft SC, then $f_{q_{1}v_{1}}^{-1}(f_{q_{2}v_{2}}^{-1}(K))=f_{\left(q_{2}\circ q_{1})(v_{2}\circ v_{1}\right)}^{-1}(K)\in {ℜ}_{\delta }$. This shows that $f_{\left(q_{2}\circ q_{1})(v_{2}\circ v_{1}\right)}$ is soft SC.

**Theorem 2.21**. *If* $f_{q_{1}v_{1}}:(Z,ℜ,E)\to (Y,P,F)$ *is a soft almost open, soft SC, and surjective, and if* $f_{q_{2}v_{2}}:(Y,P,F)\to (W,ℜ,L)$ *is a soft mapping, then* $f_{\left(q_{2}\circ q_{1})(v_{2}\circ v_{1}\right)}$ *is soft SC if and only if* $f_{q_{2}v_{2}}$ *is soft continuous*.

*Proof. Necessity.* Let $f_{\left(q_{2}\circ q_{1})(v_{2}\circ v_{1}\right)}$ be soft SC. Let *K* ∈ ℜ. Then $f_{\left(q_{2}\circ q_{1})(v_{2}\circ v_{1}\right)}^{-1}(K)=f_{q_{1}v_{1}}^{-1}(f_{q_{2}v_{2}}^{-1}(K))\in {ℜ}_{\delta }$. Since $f_{q_{1}v_{1}}$ is soft almost open, $f_{q_{1}v_{1}}(f_{q_{1}v_{1}}^{-1}(f_{q_{2}v_{2}}^{-1}(K)))\in P$. Since $f_{q_{1}v_{1}}$ is surjective, $f_{q_{1}v_{1}}(f_{q_{1}v_{1}}^{-1}(f_{q_{2}v_{2}}^{-1}(K)))=f_{q_{2}v_{2}}^{-1}(K)$. This shows that $f_{q_{2}v_{2}}$ is soft continuous.

*Sufficiency.* Let $f_{q_{2}v_{2}}$ be soft continuous. Let *K* ∈ ℜ. Then $f_{q_{2}v_{2}}^{-1}(K)\in P$. Thus, by soft super-continuity of $f_{q_{1}v_{1}}$, $f_{q_{1}v_{1}}^{-1}(f_{q_{2}v_{2}}^{-1}(K))=f_{\left(q_{2}\circ q_{1})(v_{2}\circ v_{1}\right)}^{-1}(K)\in {ℜ}_{\delta }$. This shows that $f_{\left(q_{2}\circ q_{1})(v_{2}\circ v_{1}\right)}$ is soft SC.

**Theorem 2.22**. *If* $f_{q_{1}v_{1}}:(Z,ℜ,E)\to (Y,P,F)$ *is soft almost continuous and* $f_{q_{2}v_{2}}:(Y,P,F)\to (W,ℜ,L)$ *is soft SC, then* $f_{\left(q_{2}\circ q_{1})(v_{2}\circ v_{1}\right)}$ *is soft continuous*.

*Proof*. Let $f_{q_{1}v_{1}}$ be soft almost continuous and $f_{q_{2}v_{2}}$ be soft SC. Let *K* ∈ ℜ. Since $f_{q_{2}v_{2}}$ is soft SC, $f_{q_{2}v_{2}}^{-1}(K)\in P_{\delta }$. Since $f_{q_{1}v_{1}}$ is soft almost continuous, then by Theorem 3.8(b) of [52], $f_{q_{1}v_{1}}^{-1}(f_{q_{2}v_{2}}^{-1}(K))=f_{\left(q_{2}\circ q_{1})(v_{2}\circ v_{1}\right)}^{-1}(K)\in ℜ$. This shows that $f_{\left(q_{2}\circ q_{1})(v_{2}\circ v_{1}\right)}$ is soft continuous.

The following question is natural:

**Problem 2.23**. *Let* $f_{q_{1}v_{1}}:(Z,ℜ,E)\to (Y,P,F)$ *and* $f_{q_{2}v_{2}}:(Y,P,F)\to (W,ℜ,L)$ *be two soft mappings such that* $f_{q_{1}v_{1}}$ *is soft almost continuous and* $f_{\left(q_{2}\circ q_{1})(v_{2}\circ v_{1}\right)}$ *is soft continuous. Is it true that* $f_{q_{2}v_{2}}$ *is soft SC?*

The following example gives a negative answer to Problem 2.23:

**Example 2.24**. *Let* X=R, *Y* = {*a*, *b*}, *Z* = {1, 2}, *ξ* *the co-countable topology on X*, *ϕ* = {∅, *Y*, {*a*}}, *γ* = {∅, *Z*, {2}}, *and* E=N. *Define* *q*_1_ : *X* → *Y*, *q*_2_ : *Y* → *Z*, *and* *v*_1_, *v*_2_ : *E* → *E by*

$$q_{1}\left(x\right)=\{\begin{array}{cc} a & if\;x\;is\;irrational\\ b & if\;x\;is\;rational \end{array},$$

*q*_2_(*a*) = 2, *q*_2_(*b*) = 1, *and* *v*_1_, *v*_2_ : *E* → *E* *are the identity mappings. Then q*_1_ : (*X*, *ξ*) → (*Y*, *ϕ*) *is almost continuous, and q*_2_ ∘ *q*_1_ : (*X*, *ξ*) → (*Z*, *γ*) *is continuous, but q*_2_ : (*Y*, *ϕ*) → (*Z*, *γ*) *is not SC*.

*Therefore, by Corollary 3.4 of* [44], *Theorem 5.31 of* [47], *and Corollary 2.5*, $f_{q_{1}v_{1}}:(X,\tau (\xi ),E)\to (Y,\tau (\phi ),E)$ *is soft almost continuous, and* $f_{\left(q_{2}\circ q_{1})(v_{2}\circ v_{1}\right)}$ : (*X*, *τ*(*ξ*), *E*) → (*Z*, *τ*(*γ*), *E*) *is soft continuous, but* $f_{q_{2}v_{2}}:(Y,\tau (\phi ),E)\to (Z,\tau (\gamma ),E)$ *is not soft SC*.

In Theorems 2.25, 2.27, 2.30, and Corollaries 2.26, and 2.28, we establish various preservation theorems using soft SC mappings.

**Theorem 2.25**. *Let* $f_{qv}:(Z,ℜ,E)\to (Y,P,F)$ *be soft SC. If K is a soft nearly compact set relative to* (Z,ℜ,E), *then* *f*_*qv*_(*K*) *is a soft compact subset of* (*Y*, ℘, *F*).

*Proof*. Let A⊆P such that $f_{qv}(K)\tilde{\subseteq }{\tilde{\cup }}_{A\in A}A$. Then $K\tilde{\subseteq }f_{qv}^{-1}(f_{qv}(K))\tilde{\subseteq }f_{qv}^{-1}({\tilde{\cup }}_{A\in A}A)={\tilde{\cup }}_{A\in A}f_{qv}^{-1}(A)$. Since *f*_*qv*_ is soft SC, then $\{f_{qv}^{-1}\left(A\right):A\in A\}\subseteq {ℜ}_{\delta }$. Since *K* is a soft nearly compact set relative to (Z,ℜ,E), then we find a finite subfamily $A_{1}\subseteq A$ such that $K\tilde{\subseteq }{\tilde{\cup }}_{A\in A_{1}}f_{qv}^{-1}(A)=f_{qv}^{-1}({\tilde{\cup }}_{A\in A_{1}}A)$. So, $f_{qv}(K)\tilde{\subseteq }f_{qv}(f_{qv}^{-1}({\tilde{\cup }}_{A\in A_{1}}A))\tilde{\subseteq }{\tilde{\cup }}_{A\in A_{1}}A$. This shows that *f*_*qv*_(*K*) is a soft nearly compact set relative to (*Y*, ℘, *F*).

**Corollary 2.26**. *Let* $f_{qv}:(Z,ℜ,E)\to (Y,P,F)$ *be soft SC and surjective. If* (Z,ℜ,E) *is soft nearly compact, then* (*Y*, ℘, *F*) *is soft compact*.

**Theorem 2.27**. *Let* $f_{qv}:(Z,ℜ,E)\to (Y,P,F)$ *be soft SC. If K is a soft nearly Lindelof set relative to* (Z,ℜ,E), *then f*_*qv*_(*K*) *is a soft Lindelof subset of* (*Y*, ℘, *F*).

*Proof*. Let A⊆P such that $f_{qv}(K)\tilde{\subseteq }{\tilde{\cup }}_{A\in A}A$. Then $K\tilde{\subseteq }f_{qv}^{-1}(f_{qv}(K))\tilde{\subseteq }f_{qv}^{-1}({\tilde{\cup }}_{A\in A}A)={\tilde{\cup }}_{A\in A}f_{qv}^{-1}(A)$. Since *f*_*qv*_ is soft SC, then $\{f_{qv}^{-1}\left(A\right):A\in A\}\subseteq {ℜ}_{\delta }$. Since *K* is a soft nearly Lindelof set relative to (Z,ℜ,E), then we find a countable subfamily $A_{1}\subseteq A$ such that $K\tilde{\subseteq }{\tilde{\cup }}_{A\in A_{1}}f_{qv}^{-1}(A)=f_{qv}^{-1}({\tilde{\cup }}_{A\in A_{1}}A)$. So, $f_{qv}(K)\tilde{\subseteq }f_{qv}(f_{qv}^{-1}({\tilde{\cup }}_{A\in A_{1}}A))\tilde{\subseteq }{\tilde{\cup }}_{A\in A_{1}}A$. This shows that *f*_*qv*_(*K*) is a soft nearly Lindelof set relative to (*Y*, ℘, *F*).

**Corollary 2.28**. *Let* $f_{qv}:(Z,ℜ,E)\to (Y,P,F)$ *be soft SC and surjective. If* (Z,ℜ,E) *is soft nearly Lindelof, then* (*Y*, ℘, *F*) *is soft Lindelof*.

**Theorem 2.29**. *Let* $f_{qv}:(Z,ℜ,E)\to (Y,P,F)$ *be soft SC and bijective such that* (Z,ℜ,E) *is soft nearly compact and* (*Y*, ℘, *F*) *is soft Hausdorff. Then f*_*qv*_ *is soft almost open*.

*Proof*. Let *K* ∈ ℜ_*δ*_. Then 1_*E*_ − *K* ∈ (ℜ_*δ*_)^*c*^. Since (Z,ℜ,E) is soft nearly compact, then 1_*E*_ − *K* is a soft nearly compact subset of (Z,ℜ,E). So, by Theorem 2.25, *f*_*qv*_(1_*E*_ − *K*) is a soft compact subset of (*Y*, ℘, *F*). Since (*Y*, ℘, *F*) is soft Hausdorff, then *f*_*qv*_(1_*E*_ − *K*) ∈ ℘^*c*^. Since *f*_*qv*_ is bijective, *f*_*qv*_(1_*E*_ − *K*) = 1_*F*_− *f*_*qv*_(*K*). Thus, *f*_*qv*_(*K*) ∈ ℘. This shows that *f*_*qv*_ is soft almost open.

**Theorem 2.30**. *Let* $f_{qv}:(Z,ℜ,E)\to (Y,P,F)$ *be soft SC, soft almost open, and bijective. If* (Z,ℜ,E) *is soft almost regular, then* (*Y*, ℘, *F*) *is soft regular*.

*Proof*. Let *G* ∈ ℘^*c*^ and *b*_*y*_ ∈ 1_*F*_ − *G*. Since *f*_*qv*_ is soft SC, then by Theorem 2.2(2), $f_{qv}^{-1}(G)\in {\left({ℜ}_{\delta }\right)}^{c}$. Since *f*_*qv*_ is bijective and *b*_*y*_ ∈ 1_*F*_ − *G*, then $f_{qv}^{-1}(b_{y})={\left(v^{-1}(b)\right)}_{q^{-1}(y)}\tilde{\in }f_{qv}^{-1}(1_{F}-G)=1_{E}-f_{qv}^{-1}(G)\in {ℜ}_{\delta }$. Choose H∈RO(ℜ) such that $f_{qv}^{-1}(b_{y})\tilde{\in }H\tilde{\subseteq }1_{E}-f_{qv}^{-1}(G)$. Put *S* = 1_*E*_−*H*. Then S∈RO(ℜ) with $f_{qv}^{-1}(b_{y})\tilde{\in }1_{E}-S=H\tilde{\subseteq }1_{E}-f_{qv}^{-1}(G)$. Since (Z,ℜ,E) is soft almost regular, then there are *M*, *N* ∈ ℜ such that $f_{qv}^{-1}(b_{y})\tilde{\in }M$, $f_{qv}^{-1}(G)\tilde{\subseteq }S\tilde{\subseteq }N$, and $M\tilde{\cap }N=0_{E}$. Now $M\tilde{\cap }N=0_{E}$ implies $Int_{ℜ}(Cl_{ℜ}(M))\tilde{\cap }Int_{ℜ}(Cl_{ℜ}(N))=0_{E}$. Since *f*_*qv*_ is soft almost open, then $f_{qv}(Int_{ℜ}(Cl_{ℜ}(M)))$, $f_{qv}(Int_{ℜ}(Cl_{ℜ}(N)))\in P$. Since *f*_*qv*_ is surjective, then $b_{y}=f_{qv}(f_{qv}^{-1}(b_{y}))\tilde{\in }f_{qv}(M)\tilde{\subseteq }f_{qv}(Int_{ℜ}(Cl_{ℜ}(M)))$ and $G=f_{qv}(f_{qv}^{-1}(G))\tilde{\subseteq }f_{qv}(N)\tilde{\subseteq }f_{qv}(Int_{ℜ}(Cl_{ℜ}(N)))$. Since *f*_*qv*_ is injective, then $f_{qv}(Int_{ℜ}(Cl_{ℜ}(M)))\tilde{\cap }f_{qv}(Int_{ℜ}(Cl_{ℜ}(N)))=f_{qv}(Int_{ℜ}(Cl_{ℜ}(M))\tilde{\cap }Int_{ℜ}(Cl_{ℜ}(N)))=f_{qv}(0_{E})=1_{F}$. This shows that (*Y*, ℘, *F*) is soft regular.

**Theorem 2.31**. *If* $f_{qv}:(Z,ℜ,E)\to (Y,P,F)$ *is a soft continuous mapping such that* (*Y*, ℘, *F*) *is soft regular, then f*_*qv*_ *is soft SC*.

*Proof*. Let *G* ∈ ℘. Then, by soft continuity of *f*_*qv*_, $f_{qv}^{-1}(G)\in ℜ$. To show that $f_{qv}^{-1}(G)\in {ℜ}_{\delta }$, let $e_{z}\tilde{\in }f_{qv}^{-1}(G)$. Then $f_{qv}(e_{z})\tilde{\in }G$, and by soft regularity of (*Y*, ℘, *F*), there exists *K* ∈ ℘ such that $f_{qv}(e_{z})\tilde{\in }K\tilde{\subseteq }Cl_{P}\left(K\right)\tilde{\subseteq }G$, and so, $e_{z}\tilde{\in }f_{qv}^{-1}(K)\tilde{\subseteq }f_{qv}^{-1}(Cl_{P}\left(K\right))\tilde{\subseteq }f_{qv}^{-1}(G)$. Since *f*_*qv*_ is soft continuous, we have $f_{qv}^{-1}(K)\in ℜ$ and $Cl_{P}(f_{qv}^{-1}\left(K\right))\tilde{\subseteq }f_{qv}^{-1}(Cl_{P}\left(K\right))$. Thus, we have $Int_{P}(Cl_{P}(f_{qv}^{-1}\left(K\right)))\in {ℜ}_{\delta }$ and

$$\begin{array}{ccc} e_{z} & \tilde{\in } & f_{qv}^{-1}(K)\\ & = & Int_{P}(f_{qv}^{-1}\left(K\right))\\ & \tilde{\subseteq } & Int_{P}(Cl_{P}(f_{qv}^{-1}\left(K\right)))\\ & \tilde{\subseteq } & Int_{P}((Cl_{P}(f_{qv}^{-1}\left(K\right))))\\ & \tilde{\subseteq } & Int_{P}(f_{qv}^{-1}(G))\\ & = & f_{qv}^{-1}(G)\text{.} \end{array}$$

This shows that $f_{qv}^{-1}(G)\in {ℜ}_{\delta }$. Therefore, by Theorem 2.2(5), *f*_*qv*_ is soft SC.

For any mapping *g* : *A* → *B*, the mapping *h* : *A* → *A* × *B* defined by *H*(*a*) = (*a*, *H* (*a*)) will be denoted by *g*^#^. The following result explains the relationships between a soft SC mapping and its soft graph:

**Theorem 2.32**. *Let* $f_{qv}:(Z,ℜ,E)\to (Y,P,F)$ *be a soft mapping. Then* $f_{q^{\#}v^{\#}}:(Z,ℜ,E)\to (Z\times Y,pr(ℜ\times P),E\times F)$ *is soft SC if and only if* *f*_*qv*_ *is soft SC and* (Z,ℜ,E) *is soft semi-regular*.

*Proof. Necessity.* Suppose that $f_{q^{\#}v^{\#}}$ is soft SC. Let *p* : *Z* × *Y* → *Y*, *s* : *Z* × *Y* → *X*, *u* : *E* × *F* → *E*, and *t* : *E* × *F* → *F* be the projection mappings. Then $f_{pu}:(Z\times Y,pr(ℜ\times P),E\times F)\to (Y,P,F)$ and $f_{st}:(Z\times Y,pr(ℜ\times P),E\times F)\to (Z,ℜ,E)$ are soft continuous. So, by Theorem 2.20, $f_{qv}=f_{pu}\circ f_{q^{\#}v^{\#}}$ and the soft identity mapping $f_{st}\circ f_{q^{\#}v^{\#}}:(Z,ℜ,E)\to (Z,ℜ,E)$ are soft SC. To show that (Z,ℜ,E) is soft semi-regular, we will show that $ℜ\subseteq {ℜ}_{\delta }$. Let *G* ∈ ℜ. Since $f_{st}\circ f_{q^{\#}v^{\#}=f(soq^{\#})(tov^{\#})}$ is soft SC, then $f_{\left(s\circ q^{\#})(t\circ v^{\#}\right)}^{-1}(G)=G\in {ℜ}_{\delta }$.

*Sufficiency.* Suppose that *f*_*qv*_ is soft SC and (Z,ℜ,E) is soft semi-regular. Since *f*_*qv*_ is soft SC, then *f*_*qv*_ is soft continuous. So, $f_{q^{\#}v^{\#}}$ is soft continuous. Since (Z,ℜ,E) is soft semi-regular, then by Theorem 2.17, $f_{q^{\#}v^{\#}}$ is soft SC.

**Lemma 2.33**. *Let* (Z,ℜ,E) *and* (*Y*, ℘, *F*) *be two STSs*, *A* ∈ *SS* (*Z*, *E*) − {0_*E*_}, *and B* ∈ *SS* (*Y*, *F*) − {0_*F*_}. *Then*

*(a)* A×B∈RO(pr(ℜ×P)) *if and only if* A∈RO(ℜ) *and B* ∈ *RO* (℘).
*(b)* $A\times B\in {\left(pr(ℜ\times P)\right)}_{\delta }$ *if and only if A* ∈ ℜ_*δ*_ *and B* ∈ ℘_*δ*_.

*Proof*. (a) *Necessity.* Let A×B∈RO(pr(ℜ×P)). Then

$$\begin{array}{cc} A\times B & =Int_{pr(ℜ\times P)}\left(Cl_{pr(ℜ\times P)}\left(A\times B\right)\right)\\ & =Int_{ℜ}\left(Cl_{ℜ}\left(A\right)\right)\times Int_{ℜ}\left(Cl_{ℜ}\left(A\right)\right)\text{.} \end{array}$$

Thus, $Int_{ℜ}\left(Cl_{ℜ}\left(A\right)\right)=A$ and $Int_{P}\left(Cl_{F}\left(B\right)\right)=B$. Hence, A∈RO(ℜ) and *B* ∈ *RO* (℘).

*Sufficiency.* Let A∈RO(ℜ) and *B* ∈ *RO* (℘). Then $Int_{ℜ}\left(Cl_{ℜ}\left(A\right)\right)=A$ and $Int_{P}\left(Cl_{F}\left(B\right)\right)=B$. So,

$$\begin{array}{ccc} Int_{pr(ℜ\times P)}\left(Cl_{pr(ℜ\times P)}\left(A\times B\right)\right) & = & Int_{pr(ℜ\times P)}\left(Cl_{ℜ}\left(A\right)\times Cl_{F}\left(B\right)\right)\\ & = & Int_{ℜ}\left(Cl_{ℜ}\left(A\right)\right)\times Int_{P}\left(Cl_{F}\left(A\right)\right)\\ & = & A\times B\text{.} \end{array}$$

(b) *Necessity.* Let $A\times B\in {\left(pr(ℜ\times P)\right)}_{\delta }$. Let $e_{z}\tilde{\in }A$ and $f_{y}\tilde{\in }B$. Then ${\left(e,f\right)}_{\left(z,y\right)}\tilde{\in }A\times B\in {\left(pr(ℜ\times P)\right)}_{\delta }$. So, there exists D∈RO(pr(ℜ×P)) such that ${\left(e,f\right)}_{\left(z,y\right)}\tilde{\in }D\tilde{\subseteq }A\times B$. Choose *G* ∈ ℜ and *K* ∈ ℘ such that ${\left(e,f\right)}_{\left(z,y\right)}\tilde{\in }G\times H\tilde{\subseteq }D\tilde{\subseteq }A\times B$. Thus,

$$\begin{array}{ccc} {\left(e,f\right)}_{\left(z,y\right)} & \tilde{\in } & G\times H\\ & \tilde{\subseteq } & Int_{ℜ}\left(Cl_{ℜ}\left(G\right)\right)\times Int_{P}\left(Cl_{P}\left(H\right)\right)\\ & = & Int_{pr(ℜ\times P)}\left(Cl_{pr(ℜ\times P)}\left(G\times H\right)\right)\\ & \tilde{\subseteq } & Int_{pr(ℜ\times P)}\left(Cl_{pr(ℜ\times P)}\left(D\right)\right)\\ & = & D\\ & \tilde{\subseteq } & A\times B\text{.} \end{array}$$

Therefore, $e_{z}\tilde{\in }G\tilde{\subseteq }Int_{ℜ}\left(Cl_{ℜ}\left(G\right)\right)\tilde{\subseteq }A$ and $f_{y}\tilde{\in }H\tilde{\subseteq }Int_{P}\left(Cl_{F}\left(H\right)\right)\tilde{\subseteq }B$. This shows that *A* ∈ ℜ_*δ*_ and *B* ∈ ℘_*δ*_.

*Sufficiency.* Let *A* ∈ ℜ_*δ*_ and *B* ∈ ℘_*δ*_, and let ${\left(e,f\right)}_{\left(z,y\right)}\tilde{\in }A\times B$. Then $e_{z}\tilde{\in }A$ and $f_{y}\tilde{\in }B$. Choose G∈RO(ℜ) and *H* ∈ *RO* (℘) such that $e_{z}\tilde{\in }G\tilde{\subseteq }A$ and $f_{y}\tilde{\in }H\tilde{\subseteq }B$. Thus, we have ${\left(e,f\right)}_{\left(z,y\right)}\tilde{\in }G\times H\tilde{\subseteq }A\times B$, and by (a), G×H∈RO(pr(ℜ×P)). This shows that $A\times B\in {\left(pr(ℜ\times P)\right)}_{\delta }$.

The following result shows that the soft product of two soft SC mappings is soft SC:

**Theorem 2.34**. *Let* $f_{q_{1}v_{1}}:(X,\ell ,E)\to (Y,P,F)$ *and* $f_{q_{2}v_{2}}:(Z,ℑ,S)\to (W,ℜ,T)$ be two soft mappings. Let q* : *X* × *Z* → *Y* × *W* and v* : *E* × *S* → *F* × *T* be the mappings defined by q*(*x*, *z*) = (*q*_1_(*x*), *q*_2_(*z*)) and v*(*e*, *s*) = (*v*_1_(*e*), *v*_2_(*s*)). *Then* $f_{q^{*}v^{*}}:(X\times Z,pr(\ell \times ℑ),E\times S)\to (Y\times W,pr(P\times ℜ),F\times T)$ *is soft SC if and only if* $f_{q_{1}v_{1}}$ *and* $f_{q_{2}v_{2}}$ *are soft SCs*.

*Proof. Necessity.* Suppose that *f*_q*v*_ is soft SC. Let *G* ∈ ℘ and *H* ∈ ℜ. Then G×H∈pr(P×ℜ) and so, $f_{q^{*}v^{*}}^{-1}(G\times H)=f_{q_{1}v_{1}}^{-1}\left(G\right)\times f_{q_{2}v_{2}}^{-1}(H)\in {\left(pr(\ell \times ℑ)\right)}_{\delta }$. So, by Lemma 2.33(b), $f_{q_{1}v_{1}}^{-1}\left(G\right)\in \ell_{\delta }$ and $f_{q_{2}v_{2}}^{-1}(H)\in {ℑ}_{\delta }$. This shows that $f_{q_{1}v_{1}}$ and $f_{q_{2}v_{2}}$ are soft SCs.

*Sufficiency.* Let $f_{q_{1}v_{1}}$ and $f_{q_{2}v_{2}}$ be soft SC. Let *G* ∈ ℘ and *H* ∈ ℜ. Then $f_{q_{1}v_{1}}^{-1}\left(G\right)\in {ℜ}_{\delta }$ and $f_{q_{2}v_{2}}^{-1}(H)\in {ℑ}_{\delta }$. Since $f_{q^{*}v^{*}}^{-1}(G\times H)=f_{q_{1}v_{1}}^{-1}\left(G\right)\times f_{q_{2}v_{2}}^{-1}(H)$, then by Lemma 2.33(b), $f_{q^{*}v^{*}}^{-1}(G\times H)\in {\left(pr(\ell \times ℑ)\right)}_{\delta }$. Therefore, by Theorem 2.2(6), *f*_q*v*_ is soft SC.

**Theorem 2.35**. *If* $f_{qv},f_{pu}:(Z,ℜ,E)\to (Y,P,F)$ *are soft SC mappings and* (*Y*, ℘, *F*) *is soft Hausdorff, then* $\tilde{\cup }\{e_{z}:f_{qv}(e_{z})=f_{pu}(e_{z})\}\in {\left({ℜ}_{\delta }\right)}^{c}$.

*Proof*. Let $a_{s}\tilde{\in }1_{E}-\tilde{\cup }\{e_{z}:f_{qv}(e_{z})=f_{pu}(e_{z})\}$. Then *f*_*qv*_(*e*_*z*_) ≠ *f*_*pu*_(*e*_*z*_). Since (*Y*, ℘, *F*) is soft Hausdorff, then there exist *G*, *H* ∈ ℘ such that $f_{qv}(e_{z})\tilde{\in }G$, $f_{pu}(e_{z})\tilde{\in }H$, and $G\tilde{\cap }H=0_{F}$. Since *f*_*qv*_, *f*_*pu*_ are soft SC, then $f_{qv}^{-1}(G),f_{pu}^{-1}(H)\in {ℜ}_{\delta }$.

**Claim.** $f_{qv}^{-1}(G)\tilde{\cap }f_{pu}^{-1}(H)\tilde{\cap }(\tilde{\cup }\{e_{z}:f_{qv}(e_{z})=f_{pu}(e_{z})\})=0_{E}$ .

**Proof of Claim.** Suppose, to the contrary, that there exists *b*_*t*_ such that $f_{qv}\left(b_{t}\right)\tilde{\in }G$, $f_{pu}(b_{t})\tilde{\in }H$, and *f*_*qv*_(*b*_*t*_) = *f*_*pu*_(*b*_*t*_). Then $f_{qv}(b_{t})\tilde{\in }G\tilde{\cap }H=0_{F}$, which is a contradiction.

Therefore, we have $a_{s}\tilde{\in }f_{qv}^{-1}(G)\tilde{\cap }f_{pu}^{-1}(H)\in {ℜ}_{\delta }$ and by the above claim, $f_{qv}^{-1}(G)\tilde{\cap }f_{pu}^{-1}(H)\tilde{\subseteq }1_{E}-\tilde{\cup }\{e_{z}:f_{qv}(e_{z})=f_{pu}(e_{z})\}$. This shows that 1_*E*_− $\tilde{\cup }\{e_{z}:f_{qv}(e_{z})=f_{pu}(e_{z})\}\in {ℜ}_{\delta }$. Hence, $\tilde{\cup }\{e_{z}:f_{qv}(e_{z})=f_{pu}(e_{z})\}\in {\left({ℜ}_{\delta }\right)}^{c}$.

In the following result, we discuss the behavior of soft SC mappings under soft subspaces:

**Theorem 2.36**. *Let* $f_{qv}:(Z,ℜ,E)\to (Y,P,F)$ *be a soft SC mapping. Then*

*(a) If U* ⊆ *Z* *such that* *C*_*U*_ ∈ ℜ, *then the soft restriction* ${\left(f_{qv}\right)}_{|C_{U}}:(U,{ℜ}_{U},E)\to (Y,P,F)$ *is soft SC*.
*(b) If* {*U*_*α*_ : *α* ∈ Δ} *is a cover of Z such that* $C_{U_{\alpha }}\in RO(ℜ)$ *and* ${\left(f_{qv}\right)}_{|C_{U_{\alpha }}}:(U_{\alpha },{ℜ}_{U_{\alpha }},E)\to (Y,P,F)$ *is soft SC for all α* ∈ Δ, *then* $f_{qv}:(Z,ℜ,E)\to (Y,P,F)$ *is SC*.

*Proof*. (a) Let $f_{qv}:(Z,ℜ,E)\to (Y,P,F)$ be soft SC, and let *U* ⊆ *Z* such that *C*_*U*_ ∈ ℜ. Let $e_{z}\tilde{\in }SS\left(U,E\right)$ and let *G* ∈ ℘ such that $f_{qv}(e_{z})\tilde{\in }G$. Since *f*_*qv*_ is soft SC, then there exists *H* ∈ ℜ such that $f_{qv}\left(Int_{ℜ}\left(Cl_{ℜ}\left(H\right)\right)\right)\tilde{\subseteq }G$. Then we have $H\tilde{\cap }C_{U}\in {ℜ}_{U}$, and

$$\begin{array}{ccc} {\left(f_{qv}\right)}_{|C_{U}}(Int_{{ℜ}_{U}}\left(Cl_{{ℜ}_{U}}\left(H\tilde{\cap }C_{U}\right)\right)) & = & f_{qv}(Int_{{ℜ}_{U}}\left(Cl_{{ℜ}_{U}}\left(H\tilde{\cap }C_{U}\right)\right))\\ & = & f_{qv}(C_{U}\tilde{\cap }Int_{ℜ}\left(C_{U}\tilde{\cap }Cl_{ℜ}\left(H\tilde{\cap }C_{U}\right)\right))\\ & = & f_{qv}(C_{U}\tilde{\cap }Int_{ℜ}\left(C_{U}\right)\tilde{\cap }Int_{ℜ}\left(Cl_{ℜ}\left(H\tilde{\cap }C_{U}\right)\right))\\ & = & f_{qv}(C_{U}\tilde{\cap }Int_{ℜ}\left(Cl_{ℜ}\left(H\tilde{\cap }C_{U}\right)\right))\\ & \tilde{\subseteq } & f_{qv}(Int_{ℜ}\left(Cl_{ℜ}\left(H\right)\right))\\ & \tilde{\subseteq } & G\text{.} \end{array}$$

This shows that ${\left(f_{qv}\right)}_{|C_{U}}$ is soft SC.

(b) Let {*U*_*α*_ : *α* ∈ Δ} be a cover of *Z* such that $C_{U_{\alpha }}\in RO(ℜ)$ and ${\left(f_{qv}\right)}_{|C_{U_{\alpha }}}:(U_{\alpha },{ℜ}_{U_{\alpha }},E)\to (Y,P,F)$ is soft SC for all *α* ∈ Δ. Let *G* ∈ ℘. Then $f_{qv}^{-1}(G)=f_{qv}^{-1}(G\tilde{\cap }1_{F})=f_{qv}^{-1}(G\tilde{\cap }({\tilde{\cup }}_{\alpha \in \Delta }C_{U_{\alpha }}))=f_{qv}^{-1}(({\tilde{\cup }}_{\alpha \in \Delta }(G\tilde{\cap }C_{U_{\alpha }})))={\tilde{\cup }}_{\alpha \in \Delta }f_{qv}^{-1}(G\tilde{\cap }C_{U_{\alpha }})={\tilde{\cup }}_{\alpha \in \Delta }{\left({\left(f_{qv}\right)}_{|C_{U_{\alpha }}}\right)}^{-1}(G\tilde{\cap }C_{U_{\alpha }})$. For each *α* ∈ Δ, ${\left(f_{qv}\right)}_{|C_{U_{\alpha }}}:(U_{\alpha },{ℜ}_{U_{\alpha }},E)\to (Y,P,F)$ is soft SC, and so, ${\left({\left(f_{qv}\right)}_{|C_{U_{\alpha }}}\right)}^{-1}(G\tilde{\cap }C_{U_{\alpha }})\in {\left({ℜ}_{U_{\alpha }}\right)}_{\delta }={\left({ℜ}_{\delta }\right)}_{U_{\alpha }}$. For each *α* ∈ Δ, $C_{U_{\alpha }}\in RO(ℜ)$, and so ${\left({\left(f_{qv}\right)}_{|C_{U_{\alpha }}}\right)}^{-1}(G\tilde{\cap }C_{U_{\alpha }})\in RO(ℜ)$. It follows that $f_{qv}^{-1}(G)={\tilde{\cup }}_{\alpha \in \Delta }{\left({\left(f_{qv}\right)}_{|C_{U_{\alpha }}}\right)}^{-1}(G\tilde{\cap }C_{U_{\alpha }})\in {ℜ}_{\delta }$.

## 3 Soft mappings with soft *δ*-closed graphs

In this section, we introduce several sufficient conditions for a soft mapping to have a soft *δ*-closed graph.

For a given soft mapping *f*_*qv*_ : (*Z*, *E*) → (*Y*, *T*), the soft set $\tilde{\cup }\{{\left(e,v(e)\right)}_{\left(z,q(z)\right)}:e\in E\;\text{and}\;z\in Z\}$ is called the soft graph of *f*_*qv*_ and is denoted by *G*(*f*_*qv*_). Thus, ${\left(e,t\right)}_{\left(z,y\right)}\tilde{\in }G(f_{qv})$ if and only if *f*_*qv*_(*e*_*z*_) = *t*_*y*_ if and only if *q*(*z*) = *y* and *v*(*e*) = *t*.

We start this section with a result that implies that a soft *θ*-continuous mapping takes values in a soft Hausdorff space once its values on a soft dense set are known.

**Theorem 3.1**. *If* $f_{qv}:(Z,ℜ,E)\to (Y,P,T)$ *is soft θ-continuous and* (*Y*, ℘, *T*) *is soft Hausdorff, then* $G(f_{qv})\in {\left({\left(pr(ℜ\times P)\right)}_{\delta }\right)}^{c}$.

*Proof*. Let $f_{qv}:(Z,ℜ,E)\to (Y,P,T)$ be soft *θ*-continuous, and (*Y*, ℘, *T*) is soft Hausdorff. Let ${\left(e,t\right)}_{\left(z,y\right)}\tilde{\in }1_{E\times T}-G(f_{qv})$. Then *f*_*qv*_(*e*_*z*_) ≠ *t*_*y*_. Since (*Y*, ℘, *T*) is soft Hausdorff, then there exist *G*, *H* ∈ ℘ such that $f_{qv}(e_{z})\tilde{\in }G$, $t_{y}\tilde{\in }H$, and $G\tilde{\cap }H=0_{T}$. Now, $G\tilde{\cap }H=0_{T}$ implies that $Cl_{P}\left(G\right)\tilde{\cap }Int_{P}\left(Cl_{P}\left(H\right)\right)=0_{T}$. By soft *θ*-continuity of *f*_*qv*_, there exists *K* ∈ ℜ such that $e_{z}\tilde{\in }K$ and $f_{qv}(Cl_{ℜ}\left(K\right))\tilde{\subseteq }Cl_{P}\left(G\right)$, and thus, $f_{qv}(Cl_{ℜ}\left(K\right))\tilde{\cap }Int_{P}\left(Cl_{P}\left(H\right)\right)=0_{T}$. Since $Int_{ℜ}(Cl_{ℜ}\left(K\right))\in RO(ℜ)$ and *Int*_℘_(*Cl*_℘_(*H*)) ∈ *RO* (℘), then by Lemma 2.33(a), $Int_{ℜ}(Cl_{ℜ}\left(K\right))\times Int_{P}\left(Cl_{P}\left(H\right)\right)\in RO(pr(ℜ\times P))\subseteq {\left(pr(ℜ\times P)\right)}_{\delta }$. Since $e_{z}\tilde{\in }K\tilde{\subseteq }Int_{ℜ}(Cl_{ℜ}\left(K\right))$ and $t_{y}\tilde{\in }K\tilde{\subseteq }Int_{P}\left(Cl_{P}\left(H\right)\right)$, then ${\left(e,t\right)}_{\left(z,y\right)}\tilde{\in }Int_{ℜ}(Cl_{ℜ}\left(K\right))\times Int_{P}\left(Cl_{P}\left(H\right)\right)$.

**Claim.** $(Int_{ℜ}(Cl_{ℜ}\left(K\right))\times Int_{P}\left(Cl_{P}\left(H\right)\right))\tilde{\cap }G(f)=0_{E\times T}$ .

**Proof of Claim.** Suppose, to the contrary, that there exists ${\left(a,b\right)}_{\left(z,w\right)}\tilde{\in }(Int_{ℜ}(Cl_{ℜ}\left(K\right))\times Int_{P}\left(Cl_{F}\left(H\right)\right))\tilde{\cap }G(f_{qv})$. Then $a_{z}\tilde{\in }Int_{ℜ}(Cl_{ℜ}\left(K\right))$, $b_{w}\tilde{\in }Int_{P}\left(Cl_{F}\left(H\right)\right)$, and *f*_*qv*_ (*a*_*z*_) = *b*_*w*_. Thus, $b_{w}\tilde{\in }f_{qv}(Cl_{ℜ}\left(K\right))\tilde{\cap }Int_{P}\left(Cl_{P}\left(H\right)\right)=0_{T}$, which is a contradiction.

This claim ends the proof.

**Theorem 3.2**. *For a soft mapping* $f_{qv}:(Z,ℜ,E)\to (Y,P,T)$, *the following are equivalent*:

*(a)* $G(f_{qv})\in {\left({\left(pr(ℜ\times P)\right)}_{\delta }\right)}^{c}$ .
*(b) For every* ${\left(e,t\right)}_{\left(z,y\right)}\tilde{\in }1_{E\times T}-G(f_{qv})$ , *there exist* K∈RO(ℜ) *and H* ∈ *RO* (℘) *such that* $e_{z}\tilde{\in }K$, $t_{y}\tilde{\in }H$, *and* $f_{qv}(K)\tilde{\cap }H=0_{T}$.

*Proof*. (a) → (b): ${\left(e,t\right)}_{\left(z,y\right)}\tilde{\in }1_{E\times T}-G(f_{qv})$. Since by (a), $1_{E\times T}-G(f_{qv})\in {\left(pr(ℜ\times P)\right)}_{\delta }$, there exist M∈pr(ℜ×P) such that

$$\begin{array}{ccc} {\left(e,t\right)}_{\left(z,y\right)} & \tilde{\in } & M\\ & \tilde{\subseteq } & Int_{pr(ℜ\times P)}\left(Cl_{pr(ℜ\times P)}\left(M\right)\right)\\ & \tilde{\subseteq } & 1_{E\times T}-G(f_{qv})\text{.} \end{array}$$

Choose *A* ∈ ℜ and *B* ∈ ℘ such that ${\left(e,t\right)}_{\left(z,y\right)}\tilde{\in }A\times B\tilde{\subseteq }M$. Thus,

$$\begin{array}{ccc} {\left(e,t\right)}_{\left(z,y\right)} & \tilde{\in } & A\times B\\ & \tilde{\subseteq } & Int_{ℜ}\left(Cl_{ℜ}\left(A\right)\right)\times Int_{P}\left(Cl_{P}\left(B\right)\right)\\ & = & Int_{pr(ℜ\times P)}\left(Cl_{pr(ℜ\times P)}\left(A\times B\right)\right)\\ & \tilde{\subseteq } & Int_{pr(ℜ\times P)}\left(Cl_{pr(ℜ\times P)}\left(M\right)\right)\\ & \tilde{\subseteq } & 1_{E\times T}-G(f_{qv})\text{.} \end{array}$$

Let $K=Int_{ℜ}\left(Cl_{ℜ}\left(A\right)\right)$ and *H* = *Int*_℘_(*Cl*_℘_(*B*)). Then $e_{z}\tilde{\in }K\in RO(ℜ)$ and $t_{y}\tilde{\in }H\in RO(P)$. To see that $f_{qv}(K)\tilde{\cap }H=0_{T}$, suppose, to the contrary, that there exists $a_{n}\tilde{\in }K$ such that $f_{qv}(a_{n})\tilde{\in }H$. So, we have ${\left(a,v(a)\right)}_{\left(n,q(n)\right)}\tilde{\in }G(f_{qv})$ and ${\left(a,v(a)\right)}_{\left(n,q(n)\right)}\tilde{\in }K\times H\tilde{\subseteq }1_{E\times T}-G(f_{qv})$, which is a contradiction. Therefore, $f_{qv}(K)\tilde{\cap }H=0_{T}$.

(b) → (a): Let ${\left(e,t\right)}_{\left(z,y\right)}\tilde{\in }1_{E\times T}-G(f_{qv})$. Then, by (b), there exists K∈RO(ℜ) and *H* ∈ *RO* (℘) such that $e_{z}\tilde{\in }K$, $t_{y}\tilde{\in }H$, and $f_{qv}(K)\tilde{\cap }H=0_{T}$. By Lemma 2.33(a), $K\times H\in RO(pr(ℜ\times P){)\subseteq (pr(ℜ\times P))}_{\delta }$. To show that $(K\times H)\tilde{\cap }G(f_{qv})=0_{E\times T}$, suppose, to the contrary, that there exists ${\left(a,b\right)}_{\left(n,w\right)}\tilde{\in }(K\times H)\tilde{\cap }G(f_{qv})$. Then $a_{n}\tilde{\in }K$, $b_{w}\tilde{\in }H$, and *b*_*w*_ = *f*_*qv*_ (*a*_*n*_). Thus, $b_{w}\tilde{\in }f_{qv}(K)\tilde{\cap }H=0_{T}$, which is a contradiction.

**Theorem 3.3**. *If* $f_{qv}:(Z,ℜ,E)\to (Y,P,T)$ *is soft δ-continuous and* (*Y*, ℘, *T*) *is soft Hausdorff, then* $G(f_{qv})\in {\left({\left(pr(ℜ\times P)\right)}_{\delta }\right)}^{c}$.

*Proof*. Let $f_{qv}:(Z,ℜ,E)\to (Y,P,T)$ be soft *δ*-continuous, and (*Y*, ℘, *T*) is soft Hausdorff. Let ${\left(e,t\right)}_{\left(z,y\right)}\tilde{\in }1_{E\times T}-G(f_{qv})$. Then *f*_*qv*_(*e*_*z*_) ≠ *t*_*y*_. Since (*Y*, ℘, *T*) is soft Hausdorff, then there exist *G*, *H* ∈ ℘ such that $f_{qv}(e_{z})\tilde{\in }A$, $t_{y}\tilde{\in }B$, and $A\tilde{\cap }B=0_{T}$. Now, $A\tilde{\cap }B=0_{T}$ implies that $Int_{P}(Cl_{P}\left(A\right))\tilde{\cap }Int_{P}\left(Cl_{P}\left(B\right)\right)=0_{T}$. By soft *δ*-continuity of *f*_*qv*_, there exists *K* ∈ ℜ such that $e_{z}\tilde{\in }K$ and $f_{qv}(Int_{ℜ}(Cl_{ℜ}\left(K\right)))\tilde{\subseteq }Int_{P}(Cl_{P}\left(A\right))$. Therefore, we have $e_{z}\tilde{\in }Int_{ℜ}(Cl_{ℜ}\left(K\right))\in RO(ℜ)$, $t_{y}\tilde{\in }Int_{P}\left(Cl_{P}\left(B\right)\right)\in RO(P)$, and $f_{qv}(Int_{ℜ}(Cl_{ℜ}\left(K\right)))\tilde{\cap }Int_{P}\left(Cl_{P}\left(B\right)\right)\tilde{\subseteq }Int_{P}(Cl_{P}\left(A\right))\tilde{\cap }Int_{P}\left(Cl_{P}\left(B\right)\right)=0_{T}$.

Theorem 3.2(b) ends the proof.

**Theorem 3.4**. *Let* $f_{qv}:(Z,ℜ,E)\to (Y,P,T)$ *be a soft mapping such that* $G\left(f_{qv}\right)\in {\left({\left(pr\left(ℜ\times P\right)\right)}_{\delta }\right)}^{c}$.

*(a) If L is soft nearly compact relative to* (*Y*, ℘, *T*), *then* $f_{qv}^{-1}(L)\in {\left({ℜ}_{\delta }\right)}^{c}$.
*(b) If M is soft nearly compact relative to* (Z,ℜ,E) , *then* *f*_*qv*_(*M*) ∈ (℘_*δ*_)^*c*^.
*(c) If L is a soft compact subset of* (*Y*, ℘, *T*), *then* $f_{qv}^{-1}(L)\in {\left({ℜ}_{\delta }\right)}^{c}$.
*(d) If M is a soft compact subset of* (Z,ℜ,E) , *then* *f*_*qv*_(*M*) ∈ (℘_*δ*_)^*c*^.

*Proof*. (a) Let *L* be nearly compact relative to (*Y*, ℘, *T*). We will show that $1_{E}-f_{qv}^{-1}(L)\in {ℜ}_{\delta }$. Let $e_{z}\tilde{\in }1_{E}-f_{qv}^{-1}(L)$. Then for each $t_{y}\tilde{\in }L$, ${\left(e,t\right)}_{\left(z,y\right)}\tilde{\in }1_{E\times T}-G(f_{qv})$ and by Theorem 3.2(b), there exist $K(t_{y})\in RO(ℜ)$ and *H* (*t*_*y*_) ∈ *RO* (℘) such that $e_{z}\tilde{\in }K(t_{y})$, $t_{y}\tilde{\in }H(t_{y})$, and $f_{qv}(K(t_{y}))\tilde{\cap }H(t_{y})=0_{T}$. Since *L* is soft nearly compact relative to (*Y*, ℘, *T*) and $L\tilde{\subseteq }{\tilde{\cup }}_{t_{y}\tilde{\in }L}H(t_{y})$, then there exists a finite soft subset $L_{1}\tilde{\subseteq }L$ such that $L\tilde{\subseteq }{\tilde{\cup }}_{t_{y}\tilde{\in }L_{1}}H(t_{y})$. Put $K(e_{z})={\tilde{\cap }}_{t_{y}\tilde{\in }L_{1}}K(t_{y})$. Then $e_{z}\tilde{\in }K(e_{z})\in RO(ℜ)$ and $K(e_{z})\tilde{\subseteq }1_{E}-f_{qv}^{-1}(L)$. This shows that $1_{E}-f_{qv}^{-1}(L)\in {ℜ}_{\delta }$. Hence, $f_{qv}^{-1}(L)\in {\left({ℜ}_{\delta }\right)}^{c}$.

(b) Let *M* be soft nearly compact relative to (Z,ℜ,E). We will show that 1_*T*_ − *f*_*qv*_(*M*) ∈ ℘_*δ*_. Let $t_{y}\tilde{\in }1_{T}-f_{qv}(M)$. Then for each $e_{z}\tilde{\in }M$, ${\left(e,t\right)}_{\left(z,y\right)}\tilde{\in }1_{E\times T}-G(f_{qv})$ and by Theorem 3.2(b), there exist $K(e_{z})\in RO(ℜ)$ and *H* (*e*_*z*_) ∈ *RO* (℘) such that $e_{z}\tilde{\in }K(e_{z})$, $t_{y}\tilde{\in }H(e_{z})$, and $f_{qv}(K(e_{z}))\tilde{\cap }H(e_{z})=0_{T}$. Since *M* is soft nearly compact relative to (Z,ℜ,E) and $M\tilde{\subseteq }{\tilde{\cup }}_{e_{z}\tilde{\in }M}K(e_{z})$, then there exists a finite soft subset $M_{1}\tilde{\subseteq }M$ such that $M\tilde{\subseteq }{\tilde{\cup }}_{e_{z}\tilde{\in }M_{1}}K(e_{z})$. Put $H(t_{y})={\tilde{\cap }}_{e_{z}\tilde{\in }M_{1}}K(t_{y})$. Then $t_{y}\tilde{\in }H(t_{y})\in RO(F)$ and $H(t_{y})\tilde{\subseteq }1_{T}-f_{qv}(M)$. This shows that 1_*T*_ − *f*_*qv*_(*M*) ∈ ℘_*δ*_. Hence, *f*_*qv*_(*M*) ∈ (℘_*δ*_)^*c*^.

(c) If *L* is a soft compact subset of (*Y*, ℘, *T*), then *L* is soft nearly compact relative to (*Y*, ℘, *T*) and, by (a), $f_{qv}^{-1}(L)\in {\left({ℜ}_{\delta }\right)}^{c}$.

(d) If *M* is a soft compact subset of (Z,ℜ,E), then *M* is soft nearly compact relative to (Z,ℜ,E) and, by (b), *f*_*qv*_(*M*) ∈ (℘_*δ*_)^*c*^.

**Lemma 3.5**. *If* (Z,ℜ,E) *is soft nearly compact and* L∈RC(ℜ), *then L is soft nearly compact relative to* (Z,ℜ,E).

*Proof.* Let (Z,ℜ,E) be soft nearly compact, and L∈RC(ℜ). Let H⊆RO(Z,ℜ,E) such that $L\tilde{\subseteq }{\tilde{\cup }}_{H\in H}H$. Let $H_{1}=H\cup \{1_{E}-L\}$. Then $H_{1}\subseteq RO(ℜ)$ and $1_{E}={\tilde{\cup }}_{H\in H_{1}}H$. Since (Z,ℜ,E) is soft nearly compact, there exists a finite subcollection $H_{2}\subseteq H_{1}$ such that $1_{E}={\tilde{\cup }}_{H\in H_{2}}H$. Let $H_{3}=H_{2}-\{1_{E}-L\}$. Then $H_{3}$ is a finite subcollection of H such that $L\tilde{\subseteq }{\tilde{\cup }}_{H\in H_{3}}H$. This shows that *L* is soft nearly compact relative to (Z,ℜ,E).

**Theorem 3.6**. *If* $f_{qv}:(Z,ℜ,E)\to (Y,P,T)$ *is a soft mapping such that* $G(f_{qv})\in {\left({\left(pr(ℜ\times P)\right)}_{\delta }\right)}^{c}$ *and* (*Y*, ℘, *T*) *is soft nearly compact, then f*_*qv*_ *is soft δ-continuous*.

*Proof*. Let *L* ∈ *RC*(℘). Since (*Y*, ℘, *T*) is soft nearly compact, by Lemma 3.5, *L* is soft nearly compact relative to (*Y*, ℘, *T*). So, by Theorem 3.4(a), $f_{qv}^{-1}(L)\in {\left({ℜ}_{\delta }\right)}^{c}$. Therefore, by Theorem 6.2(8) of [30], *f*_*qv*_ is soft *δ*-continuous.

**Theorem 3.7**. *If* $f_{qv}:(Z,ℜ,E)\to (Y,P,T)$ *is a soft mapping such that* $G(f_{qv})\in {\left({\left(pr(ℜ\times P)\right)}_{\delta }\right)}^{c}$ *and* (*Y*, ℘, *T*) *is soft compact, then* *f*_*qv*_ *is soft SC*.

*Proof*. Let *L* ∈ ℘^*c*^. Since (*Y*, ℘, *T*) is soft compact, then *L* is a soft compact subset of (*Y*, ℘, *T*). So, by Theorem 3.4(c), $f_{qv}^{-1}(L)\in {\left({ℜ}_{\delta }\right)}^{c}$. Therefore, by Theorem 2.2(2), *f*_*qv*_ is soft SC.

**Theorem 3.8**. *If* $f_{qv}:(Z,ℜ,E)\to (Y,P,T)$ *is soft* *δ-continuous and M is soft nearly compact relative to* (Z,ℜ,E), *then f*_*qv*_ (*M*) *is soft nearly compact relative to* (*Y*, ℘, *T*).

*Proof*. Let $f_{qv}:(Z,ℜ,E)\to (Y,P,T)$ is soft *δ*-continuous and *M* is soft nearly compact relative to (Z,ℜ,E). Let K⊆RO(P) such that $f_{qv}(M)\tilde{\subseteq }{\tilde{\cup }}_{K\in K}K$. Then $f_{qv}^{-1}(f_{qv}(M))\tilde{\subseteq }f_{qv}^{-1}({\tilde{\cup }}_{K\in K}K)={\tilde{\cup }}_{K\in K}f_{qv}^{-1}(K)$. Since *f*_*qv*_ is soft *δ*-continuous, then by Theorem 6.2(7) of [30], $\left\{f_{qv}^{-1}(K):K\in K\}\subseteq RO(Z,ℜ,E\right)$. Since *M* is soft nearly compact relative to (Z,ℜ,E), then there exists a finite subcollection $K_{1}\subseteq K$ such that $M\tilde{\subseteq }{\tilde{\cup }}_{K\in K_{1}}f_{qv}^{-1}(K)$. Thus, $f_{qv}(M)\tilde{\subseteq }{\tilde{\cup }}_{K\in K_{1}}f_{qv}^{-1}(K)=f_{qv}(f_{qv}^{-1}({\tilde{\cup }}_{K\in K_{1}}K))\tilde{\subseteq }{\tilde{\cup }}_{K\in K_{1}}K$. This shows that *f*_*qv*_ (*M*) is soft nearly compact relative to (*Y*, ℘, *T*).

**Corollary 3.9**. *Soft near compactness is preserved under soft* *δ*-*continuous surjections*.

## 4 Conclusion

Numerous aspects of everyday life are uncertain. The soft set theory is one idea put out to deal with uncertainty. This work focuses on soft topology, a special mathematical framework that topologists have created by using soft sets.

The uncertain versions of topology, like soft topology, are vital tools to transact with many impediments that we face in different situations of our lives; this matter can be noted from the published manuscripts that exploited topological concepts such as compactness, separation axioms, and generalizations of open sets to address these situations as illustrated [21, 59, 60].

In this paper, soft super-continuity is defined as a new type of soft mapping that is a strong form of each of soft continuity and soft *δ*-continuity and a weaker form of soft complete continuity. Several characterizations (Theorems 2.2, 2.17), relationships (Theorems 2.3, 2.4, 2.6, 2.8, 2.13, 2.15, 3.6, 3.7, Examples 2.7, 2.9, 2.14, Corollary 2.16), compositions (Theorems 2.20, 2.21, 2.22, Example 2.24), restrictions (Theorem 2.36), preservations (Theorems 2.25, 2.27, 2.30, 3.4, 3.8, Corollaries 2.26, 2.28, 3.9), and products (Theorems 2.32, 2.34) of soft super-continuity are introduced. In addition, the relationships between soft super-continuity and soft *δ*-continuity and their analogous notions in general, topology are studied (Theorems 2.3, 2.4, 2.10, 2.11, Corollaries 2.5, 2.12). Finally, several sufficient conditions for the soft mapping to have a soft *δ*-closed graph are given (Theorems 3.1, 3.2, 3.3).

Soft super-continuous functions are anticipated to find use in several fields, including robotics, soft data analysis, soft image processing, and soft decision-making. They offer a framework for representing and evaluating ambiguous and imprecise data, resulting in algorithms and systems that are more adaptable and versatile.

In the future, we may look at the following topics: (1) studying further properties of soft super-continuous functions; (2) defining soft strongly super-continuous mappings; and (3) finding an application for our new results in the “decision-making problem,” “information systems,” or “expert systems”.
